# Herpes and Brachial Neuritis

**Published:** 1911-12

**Authors:** G. Munro Smith

**Affiliations:** Consulting Surgeon, Bristol Royal Infirmary


### HERPES AND BRACHIAL NEURITIS.

BY

G. MUNRO SMITH,

*Consulting Surgeon, Bristol Royal Infirmary.*

I SHOULD like to preface this short communication by a fragment of personal history.

On April 21st, 1910, I awoke in the morning with pain in my left arm and shoulder, becoming very severe on movement, so that dressing was a great difficulty. For a few days before this I had experienced occasional slight pains in my limbs, which I thought were rheumatic; and the day before (April 20th) I had gone a long country ramble, which perhaps slightly tired me. I went on with my work for four days in great misery, and then succumbed, and was laid up for a month with what was soon recognised to be an attack of brachial neuritis. During the early part of the attack I could only get comparative freedom from pain by lying on my face or on all fours, and I actually spent the night in the latter position more than once. The pain, which never really left me, settled down into two severe exacerbations every night, one between 1 and 2 a.m. and the other between 3 and 5 a.m. I took chloral, veronal, and once morphia. I also took various other drugs; but nothing did me any real good except very gentle massage, warm bathing and diversion from the nocturnal pain by tea, which I took every night twice during my illness. During the attacks of pain my temperature rose to 100°, 100.5°, sometimes 101°. I had occasional slight œdema of the left hand, paræsthesia of radial side of the thumb, and hyperæsthesia of the whole of the left hand. The part affected was chiefly the root of the 7th cervical, but the inflammation was not strictly confined to this, and during my recovery the vaso-motor phenomena, the heat, redness, cyanosis and feeling of weight involved to some extent the right arm and very slightly the left foot.

Several medical friends were kind enough to see me, and even prescribed for me ; but I felt much in the mood of Montaigne, who said, " I answer such as urge me to take Physicke that at least they will tarry till such times as I have recovered my health and strength againe : that then I may the better be enabled to endure the violence and hazard of their potions."

When one is brought into contact with a malady in one's own person one generally comes across similar cases, so I was not surprised to hear from several of my friends and patients that they had suffered from brachial and other forms of neuritis. But in the latter part of the year I saw two real cases of brachial neuritis—one in an elderly woman and one in a middle-aged man. Soon after a patient of mine had a typical attack of the complaint. When I say typical, I may perhaps explain my meaning by saying that it conformed to the description by Gowers, which is one of the best accounts of the disease I know. In November I had a case in a young woman involving the circumflex and branches of the musculo-spiral. In February of this year I saw a young married woman with brachial neuritis. The same month I saw a servant-maid with the same. On April 14th I saw a lady, aged 65, who was taken ill at Naples on April 6th with temperature, extreme drowsiness and slight intestinal disturbance. On April 7th her right arm became affected, chiefly branches of the ulnar. Besides these I heard of other cases which I did not see.

In October, 1910, I saw an old lady with severe herpes frontalis. On January 21st of this year I saw a lady, aged about 33, with herpes zoster. She suffered very severe pain, especially at night, and spent two or three nights walking about, restless with pain.

On January 25th I saw a young man, aged 25, with the same complaint, and on February 6th I saw a nurse, aged 40, with bad herpes zoster. Her pain was very severe, but was partially controlled by full doses of chloral and bromide.

Towards the end of April one of the cases I have referred to as ulnar neuritis developed redness and hyperæmia on the hypothenar eminence and three or four very minute vesicles.

I heard of several other cases. In fact, there could be no doubt that there was a prevalence of herpes, especially herpes zoster; and it is a well-known fact that herpes zoster occurs in epidemics. Accounts came from various parts of England of similar epidemics. Dr. George Parker and others recorded the prevalence also of "anterior poliomyelitis."

In an excellent article on the pathology of herpes zoster, by Drs. Head and Campbell, in *Brain* for 1900, Part III., the close similarity, pathologically, of these two diseases is forcibly pointed out, the authors contending, and I think with convincing arguments, and proofs derived from *post-mortem* examinations, that herpes zoster is a disease, probably microbial in origin, of the posterior root ganglia; that it is, in fact, a "posterior poliomyelitis," the ganglia corresponding physiologically with the anterior grey cornua.

If it be granted that this is so, may we not go a step farther and include brachial neuritis in the same category as an infective disease of the posterior nerve ganglia? The fact that both diseases have a distribution pointing to a radicular and not a branch origin in so many cases is in itself a strong argument as to their pathological affinity. Then, again, the preceding *malaise*, sometimes very slight, but frequently present; the great pain, the loss of sensation or paræsthesia during and following the attack; the occasional rise of temperature, the apparent occurrence of both complaints in outbreaks or epidemics, and the spreading of the original lesion to other neighbouring parts of the nervous system, etc., all point to a strong similarity. The fact that the posterior ganglia are more isolated than the nerve cells of the anterior cornua would limit the lesion more in these cases than in anterior poliomyelitis.

As to the method by which the infection enters the system, it seems likely that this in both cases may be the gastro-intestinal tract. In two of my cases there was considerable disturbance of digestion before the attack, and it is of interest perhaps to note that in my own case any indigestion seemed invariably to increase the severity of the pain.

I am aware that this idea of the pathological identity of herpes zoster and brachial neuritis is only a speculation on my part. I have no knowledge of nervous diseases to justify me in bringing it forward except as a suggestion, the outcome of that greater interest in a malady induced by painful personal experience.

---

### THE BRISTOL MEDICAL LIBRARY.

